# Evaluation of the effects of adding an adipose tissue‐derived stromal vascular fraction to platelet‐rich plasma injection in the treatment of androgenetic alopecia: A randomized clinical trial

**DOI:** 10.1111/srt.13700

**Published:** 2024-04-17

**Authors:** Elham Behrangi, Seyyedeh Tahereh Rahimi, Sona Zare, Azadeh Goodarzi, Mohammadreza Ghassemi, Fariba Khodadad, Maryam Nouri, Samaneh Mozafarpoor, Abbas Dehghani, Mohammad Ali Nilforoushzadeh, Masoumeh Roohaninasab

**Affiliations:** ^1^ Department of Dermatology Rasool Akram Medical Complex Clinical Research Development Center (RCRDC) School of Medicine Iran University of Medical Sciences Tehran Iran; ^2^ Skin and Stem Cell Research Center Tehran University of Medical Sciences Tehran Iran; ^3^ Laser Application in Medical Sciences Research Center Shahid Beheshti University of Medical Sciences Tehran Iran; ^4^ Stem Cell and Regenerative Medicine Center Sharif University of Technology Tehran Iran; ^5^ Department of Mechanical Engineering Sharif University of Technology Tehran Iran; ^6^ Department of Dermatology Faculty of Medicine Isfahan university of Medical Sciences Isfahan Iran; ^7^ Skin Repair Research Center Jordan Dermatology and Hair Transplantation Center Tehran Iran

**Keywords:** androgenetic alopecia, hair loss, platelet‐rich plasma, randomized clinical trial, stromal vascular fraction

## Abstract

**Background:**

Stromal vascular fraction (SVF) cells derived from adipose tissue and platelet‐rich plasma (PRP) are among novel treatments for androgenetic alopecia (AGA). We aimed to investigate the effect of adding SVF to PRP and compare it to administering PRP injection alone.

**Methods:**

Eighteen patients were randomly divided into two groups of nine. The PRP group was treated with PRP at all three visits at 1‐month intervals, while the SVF‐PRP group received an SVF injection on the first visit and a PRP injection on the second and third visits. Each group was evaluated at baseline and 20 weeks after the therapy's initiation.

**Results:**

Changes in mean hair diameter and hair count compared to baseline were significant in both groups. The PRP group experienced a greater increase in mean hair count than the SVF‐PRP group, and the SVF‐PRP group had a marginally greater increase in hair diameter than the PRP group. These differences were not statistically significant compared to each other. The patient and physician assessment scores exceeded the mean (on a scale from 0: poor to 3: excellent) in both groups.

**Conclusion:**

Adding one SVF injection to two PRP treatment sessions versus three PRP injections alone had no significant difference in evaluated variables. If additional research demonstrates the same results, we suggest that multiple SVF injection sessions may be required to produce a statistically significant difference compared to PRP injection alone. Moreover, considering lower cost and greater accessibility of PRP, it can be used before SVF in the treatment of AGA.

## 1 INTRODUCTION

Given the high prevalence of androgenetic alopecia (AGA), its significant impact on the patient's satisfaction with their appearance, and the possible link between chronic baldness and skin cancers, researchers have always been interested in exploring the most effective treatment for this type of hair loss.^1^, ^2^, ^3^ FDA‐approved treatments (finasteride 1 mg in MPHL and topical minoxidil in FPHL/MPHL), off‐label treatments (e.g., finasteride 5 mg, dutasteride 0.5 mg), emerging treatments (low‐level laser therapy, Botulinum toxin, and others), and hair transplants are among the options currently available.^4^

In topical and oral treatments, the long duration of use and side effects, and in hair transplantation, the high cost and limitations in patient selection criteria must be considered.^5^ Studies on novel AGA treatment methods with shorter duration and fewer side effects have increased in recent years. Examples of these methods include stromal vascular fraction (SVF) and platelet‐rich plasma (PRP).

PRP consists of a high concentration of various growth factors released from the alpha granules of platelets^6^, ^7^ and is prepared from the patient's blood either manually or using commercially available kits. SVF is a heterogeneous group of mature, progenitor, and ancestral cells that can have a regenerative and anti‐inflammatory effect with the ability to secrete cytokines^8^ and are prepared by enzymatic or mechanical means from the patient's harvested fat. To better understand the potential effect of SVF and PRP on AGA, a brief overview of the pathogenesis of AGA is provided. Concerning the polygenic inheritance of AGA, genes related to androgen receptors (AR genes), genes associated with WNT signaling, and the aromatase gene (in FPHL) are several genes presumed to be involved in its pathogenesis.^9^

Under the influence of genetics, hormones, and environmental factors, the anagen phase is shortened, whereas the telogen phase remains constant or lengthens. As the process of anagen phase shortening and telogen phase lengthening (shortening of the hair growth cycle) continues, terminal hair undergoes a process called miniaturization to become vellus.^8^ Despite the clinical differences between FPHL and MPHL, the histological hallmark of both is the miniaturization of hair follicles.^10^

The interaction of hair follicular stem cell (HFSC) and HFSC niche is necessary for the cyclic growth of hair, and in AGA, the disruption of dermal papillae cells (DPCs) causes defects in the path of this interaction, thereby affecting hair growth.^11^ The changes of DPCs in AGA include increased placement of the nuclear receptor of androgens, microvascular changes in the form of apoptosis of the endothelial cells of micro dermal papilla vessels, increased secretion of Dkk1, which is a negative regulator of WNT signaling, upregulation of transforming growth factor beta (TGF‐ β1), which results in the transition of the anagen phase to the catagen phase, and the increase of inflammatory cytokines including IL‐6, which leads to inhibiting the entry into the anagen phase and disrupting its normal progression.^11^ Moreover, AGA is significantly influenced by the androgen receptor pathway, WNT signaling, and apoptosis.^12^

PRP functions via the upregulation of β‐catenin and fibroblast growth factor‐7 (FGF‐7) signaling, as well as anti‐apoptotic effects (Bcl2 release).^12^, ^13^ SVF secretes insulin‐like growth factor 1 (IGF‐1), platelet‑derived growth factor (PDGF), and anti‐inflammatory cytokines. Thus, SVF and PRP are assumed to stimulate hair follicle proliferation and inhibit apoptosis.^8^ This clinical trial evaluates the efficacy, patient satisfaction, and side effects of adding SVF to PRP injection versus using PRP alone to treat AGA.

## 2 MATERIALS AND METHODS

### 2.1 Patients

A total of 18 eligible patients were selected from those referred to the skin clinic of the hospital where the authors affiliated to with a diagnosis of AGA from April to September 2021 and were randomly divided into two groups of nine using a table of random numbers.

The inclusion criteria included males and females diagnosed with AGA, between 18 and 60 years old, Hamilton score of 2–4 in males and Ludwig score of 1–3 in females, and full patient consent to participate in the study. The exclusion criteria included platelet disorders, thrombocytopenia, receiving anticoagulant drugs, malignancies, chemotherapy over the last 5 years, sepsis, smoking, pregnancy, wound or active infection at the treatment site, topical or systemic hair loss medications used during the previous 3 months, and females with hyperprolactinemia, hormonal disorders, or polycystic ovaries.

A full explanation of the treatment process was provided at baseline, and written consent was obtained from all participants. The study was assessed and approved by the ethics committee. Moreover, the study complied with the Declaration of Helsinki.

### 2.2 SVF cells isolation

Under sterile conditions, after local anesthesia with the injection of a tumescent solution, 20–30 mL of fat was harvested from the lower abdomen or thigh using a handheld cannula. Afterward, blood was slowly drained from the syringe, and fat tissue was transferred from the syringe to a 50‐mL Falcon tube to prepare SVF from the harvested fat. The tube was then inverted multiple times and centrifuged at 1000 revolutions per minute (RPM) for 5 min. The supernatant was subsequently discarded, and an equal volume of 0.1 mg/mL collagenase was added to the tube and incubated for 60 min. Then, the enzyme was neutralized by adding an equal volume of culture medium containing 10% fetal bovine serum (FBS), followed by pipetting for 5 min. The solution was then transferred to 1‐mL syringes for injection. Finally, the harvest site was dressed with a pressure dressing, and the patients were discharged with oral prophylactic antibiotics.

### 2.3 PRP preparation

First, 20 mL of blood was extracted from the cubital vein, followed by the addition of an anticoagulant (citrate dextrose dilution) at a ratio of 1:10 (1 mL of anticoagulant per 10 mL of patient blood). After a 10‐s wait to ensure that the blood and anticoagulant were thoroughly mixed, the tubes containing the compound were centrifuged for 5 min at 1500 RPM. After centrifugation, three parts formed in the tube: platelet‐poor plasma (PPP), a buffy coat, and a part containing the red blood cells. The part containing PPP and buffy coat was centrifuged at 1200 RPM for 5 min to obtain the final concentration. Approximately 6 mL of the obtained PRP was injected using 1‐mL syringes.

### 2.4 SVF and PRP injection

There were three treatment sessions at 1‐month intervals for both groups. In the PRP group, PRP was injected at each visit. In the SVF‐PRP group, SVF was administered at the initial visit, while PRP was administered at subsequent appointments. The area was sterilized with a cotton alcohol swab before injection, and 2% lidocaine was injected locally to achieve ring‐block anesthesia. In both groups, intradermal injections were administered to affected areas of the scalp using 1‐mL syringes with 30‐gauge needles, with injection points approximately 1 cm apart. Patients were instructed not to cleanse their scalps for 24 h.

### 2.5 Assessment

At baseline, a questionnaire including age, gender, alopecia grade, and others was completed. Visio Face was used to photograph the scalp of each patient. A basic trichoscopy image (utilizing a KC technology hair polarizer) was acquired from a particular scalp region to calculate the hair count by a ×60 lens and the average hair diameter through a ×150 lens in millimeters in that region. Both groups were followed up 2 months after the last treatment session (i.e., 5 months after the start of treatment). Once again, a Visio Face image of the patient's scalp was taken in a similar position to the baseline image. The image was shown to a physician blinded to the type of treatment used to obtain the physician's assessment score based on the improvements seen in the images after treatment (poor = 0 points, acceptable = 1 point, good = 2 points, and excellent = 3 points).

Each patient had a TrichoScan performed in the same area as the baseline, and the hair count and average hair diameter were recorded for comparison purposes. In the follow‐up session, the patient's satisfaction with the treatment process was recorded using a patient assessment score (from 0 to 3, similar to the physician's assessment score).

## 3 RESULTS

Two months after the last treatment session, all patients completed treatment and follow‐up appointments (5 months after the start of treatment). No severe complications were reported during the treatment and follow‐up of the patients, and the most common complaints were pain at the injection site and headache. Table 1 displays the demographic data of the participants in the study.

**TABLE 1:** Demographic statics of the patients.

Variable		Total	Group		*p*‐value
			PRP	SVF PRP	
Age		40.44 ± 11.15	40.67 ± 10.61	40.22 ± 12.31	0.936
Gender	Male	5 (27.8%)	1 (11.10%)	4 (44.40%)	0.294
	Female	13 (72.2%)	8 (88.90%)	5 (55.60%)	
Hamilton Norwood Scale	2	2 (40.0%)	–	2 (50.0%)	1.00
	3	3 (60.0%)	1 (100%)	2 (50.0%)	
Ludwig Scale	2	4 (33.3%)	3 (42.9%)	1 (20.0%)	0.576
	3	8 (66.7%)	4 (57.1%)	4 (80.0%)	

The mean hair count in the PRP group was 15.33 ± 3.12 before and 26.78 ± 5.14 after injection. In other words, after receiving the injection, the mean hair count increased by 11.44 ± 4.69 (*p* < 0.001). In addition, the mean hair diameter before and after injection was 0.06 ± 0.02 and 0.09 ± 0.02, indicating an increase of 0.03 ± 0.02 after injection (*p* < 0.001).

In the SVF‐PRP group, the mean hair count before and after injection was 18.67 ± 2.78 and 26.44 ± 4.19, respectively. In other words, the mean hair count increased by 7.78 ± 3.03 after the injection, which was statistically significant (*p* < 0.001). In addition, the mean hair diameter before and after injection was 0.05 ± 0.01 and 0.08 ± 0.01, respectively, indicating an increase of 0.03 ± 0.01 (*p* < 0.001) after injection.

**FIGURE 1:**
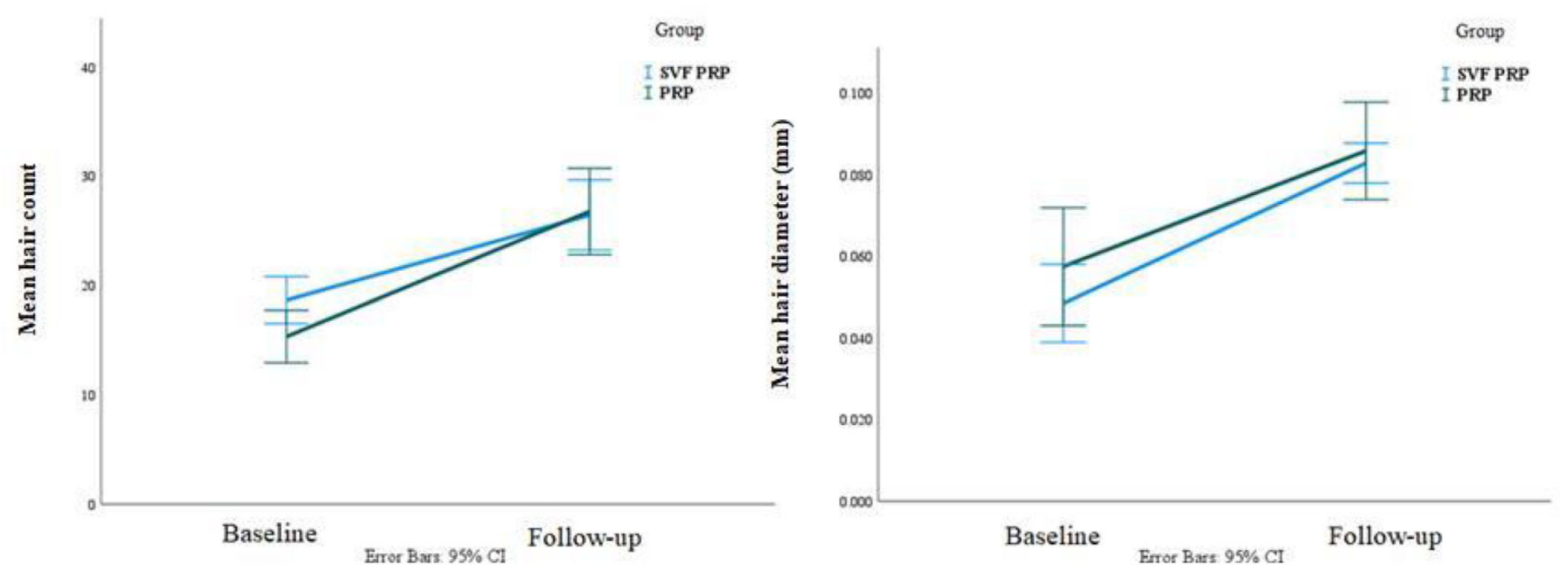
Comparison of mean hair count (left) and mean hair diameter (right) between PRP and SVF‐PRP group.

By controlling the hair count before injection, the mean hair count in the SVF‐PRP group was 3.21 units lower than in the PRP group; however, this difference was not statistically significant (*p* = 0.917). In addition, by controlling hair diameter before injection, the difference in mean hair diameter between the SVF‐PRP and PRP groups was negligible (*p* > 0.001), and the difference was not statistically significant (*p* = 0.170). The test power for the group variable's hair count and diameter was 0.05 and 0.27, respectively (Figure 1).

**FIGURE 2:**
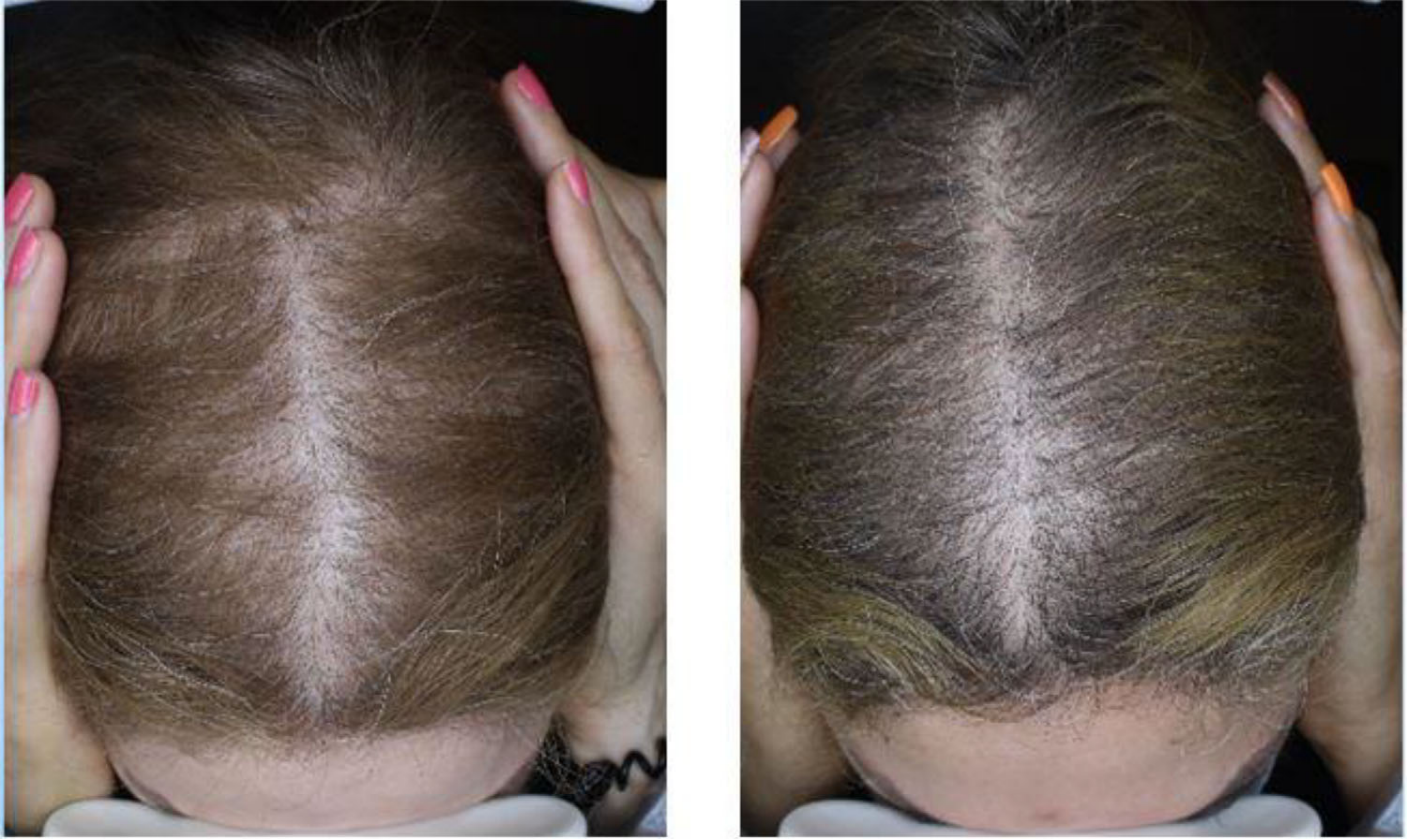
Baseline (left) and follow‐up (right) pictures of a 45 years old woman in PRP group.

**FIGURE 3:**
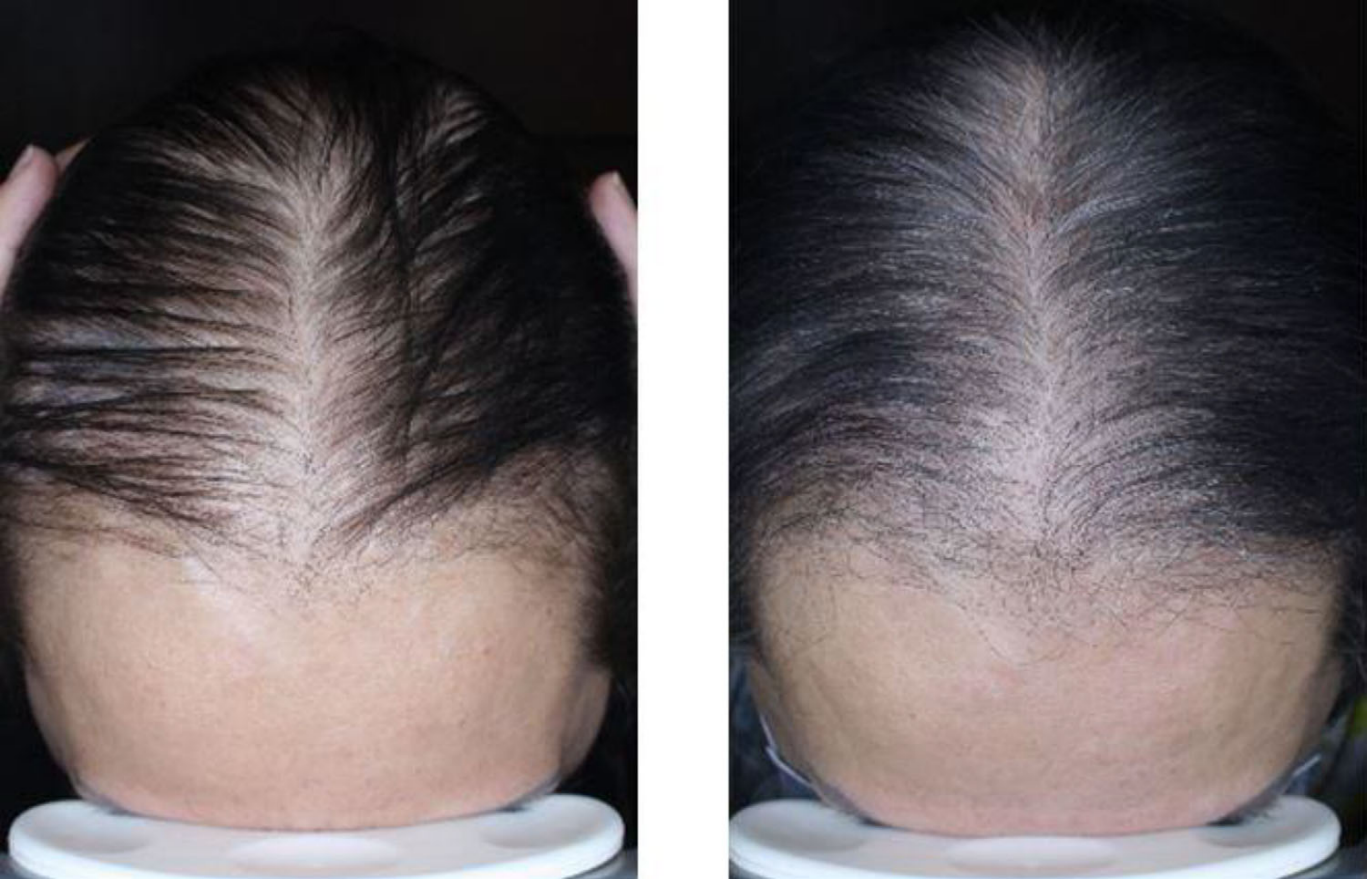
Baseline (left) and follow‐up (right) pictures of a 52 years old woman in SVF‐PRP group.

The mean patient assessment scores for the PRP and SVF‐PRP groups were 2.11 ± 0.60 and 2.22 ± 0.67, respectively. The mean physician assessment score for the PRP group (Figure 2) was 2.22 ± 0.67 and 2.33 ± 0.71 in the SVF‐PRP group (Figure 3).

The average of the patient and physician assessment scores, which were ordinal scores ranging from 0 to 3, was 1.5. This indicates that the scores obtained for patient and physician satisfaction were above average (Table 2).

**TABLE 2:** Comparison of patient and physician satisfaction.

	Patient assessment score		Physician assessment score	
Group type	Mean ± SD	*p*‐value	Mean ± SD	*p*‐value
**PRP**	2.11 ± 0.60	0.627	2.22 ± 0.67	0.657
**SVF‐PRP**	2.22 ± 0.67		2.33 ± 0.71	

## 4 DISCUSSION

Previous studies demonstrated autologous PRP's beneficial effects on AGA patients’ hair regrowth. Review and meta‐analysis studies revealed a significant increase in hair density and diameter compared to the baseline/control group.^14^, ^15^, ^16^ Similar results were observed in the present study, and the PRP group demonstrated a significant increase in hair density and diameter.

Concerning the relationship between age and gender and the effect of PRP, Georgescu et al. observed that changes in hair density were significantly inversely correlated with age and unrelated to gender.^16^ Furthermore, Gentile et al. found that the treatment was more effective on men with less severe alopecia.^17^ In the present study, there was no correlation between gender and age and treatment outcomes. This lack of significance is likely attributable to the small sample size.

There is currently no standard protocol for PRP preparation, injection intervals, the total number of treatment sessions, and volume injected. Georgescu et al. did not observe a significant increase in hair density with more total treatment sessions and a larger volume of PRP injections, but they did observe a significant increase with more sessions per month.^16^ Gentile et al.,^17^ Stevens et al.,^18^ and Sharma et al.^19^ suggested, for AGA treatment, a minimum of three to five sessions with a 1‐month interval, a minimum of six sessions per year (consisting of three sessions monthly followed by three seasonal sessions), and a minimum of three to five sessions with a 1‐month interval, respectively. Gkini et al. considered re‐injection after 6 months necessary to maintain and improve PRP's positive results.^20^

Concerning PRP preparation technique and activator addition, Gentile et al. deemed both active and inactive autologous PRP effective.^17^ Stevens et al.^18^ recommended using an activator, while El‐Husseiny et al. did not observe a more significant effect of adding an activator.^21^

Regarding centrifugation, Stevens et al. recommended single‐spin centrifugation.^18^ In their study of 15 patients, El‐Husseiny et al.^21^ treated the right side of the scalp with PRP and double‐spin centrifugation, while the left side was treated with PRP and single‐spin centrifugation. Injections were administered in three sessions spaced 3 weeks apart, followed by an examination 6 weeks after the final session. An increase in hair density was observed in both groups. Still, this increase was significant in half treated with double‐spin PRP compared to single‐spin, and the researchers concluded that double‐spin centrifugation is more effective for PRP preparation.

Based on substantial evidence, Sharma et al.^19^ concluded that the manual method with double‐spin is the preferred method for preparing PRP and did not consider using an activator necessary. The present study extracted PRP manually using a double‐spin centrifuge without an activator.

Kang et al. examined the safety and efficacy of SVF derived from autologous adipose tissue for treating non‐scarring hair loss in a systematic literature review up to November 2020. This study indicated that SVF could effectively treat non‐scarring hair loss without serious complications. The study observed that the extent of the effectiveness of this treatment potentially depends on many variables, including the severity and underlying cause of the patient's alopecia, frequency of treatment, concomitant treatments, and preparation methods.^22^

In their study of nine patients treated with a single session of SVF injection, Kim et al. observed a significant increase in hair density after 3 and 6 months of follow‐up and an increase in hair thickness and scalp keratin score after 6 months.^23^

In the study by Öztürk et al., 20 patients received a single SVF injection and were evaluated 3 months later. They reported satisfactory changes in total hair density and thickness, particularly in the bitemporal region. Nevertheless, there was no change in the hair density of 25% of males in the temporoparietal area and the hair thickness in the vertex in half of the patients. Furthermore, their study did not include the initial and final hair density and thickness measurements, and the changes were expressed as percentages.^24^

The quality of SVF depends on the method and place of fat harvesting, the patient's characteristics, and the method of SVF preparation. Despite the many differences in fat harvesting and SVF preparation methods, there is still no standard method for its preparation. SVF is prepared manually or using automatic and semi‐automatic machines from the fat isolated by enzymatic or mechanical techniques. Automatic devices reduce the risk of infection by creating a sterile environment, are less dependent on the operator, and are more expensive than manual methods. The mechanical approach involves mechanical stirring to destroy the collagen of the extracellular matrix and release SVF.

The enzymatic method includes rinsing and enzymatic digestion (such as collagenase, trypsin, and others), centrifugation with or without gravity separation and filtration, and centrifugation with or without gravity separation. Comparisons between the mechanical and enzymatic methods have failed to demonstrate that the mechanical method is as effective as the enzymatic method, despite the mechanical method being more cost‐ and time‐efficient.^8^ In this study, adipose‐derived SVF was isolated manually using an enzymatic method.

In a study by Butt et al., 11 patients received SVF and PRP injections in two sessions spaced 1 month apart, and 11 patients received PRP injections alone. Six months after the last session, the hair density and hair pull test assessment was performed, although hair diameter was not reported. The mean hair density increased from 52.44 ± 9.66 to 63.72 ± 7.73 in the PRP group and from 37.66 ± 7.43 to 57.11 ± 7.73 in the SVF and PRP groups. Compared to the PRP group, hair density was significantly increased in the PRP and SVF combination group. Since both groups experienced an increase in hair density, the authors recommend using PRP in the early stages of AGA and a combination of SVF and PRP in more severe cases. The mean number of hairs pulled in both groups decreased, but the reduction was more significant in the SVF and PRP groups. The authors suggested that SVF may be a viable option for patients with hair loss as their chief complaint. The physician and patient satisfaction scores were compared after 1 and 6 months of treatment. Both groups showed improved assessment scores, but the SVF‐PRP group's improvement was more significant.^25^

However, in the present study, both patient and physician assessment scores were above average, but the difference between the two groups was not statistically significant. In addition, the mean hair count increased significantly in both groups, but the difference between the PRP and SVF‐PRP groups was not statistically significant. The number of SVF injection sessions is one of the possible explanations for this difference (one SVF injection added to PRP in the present study versus two injections in Butt et al.). Although multiple studies^23^, ^26^ have demonstrated the efficacy of a single treatment session with SVF in improving AGA, it can be hypothesized that for SVF to have a significant effect compared to the baseline, one treatment session can be effective, but for it to be more effective than PRP, it should be injected in multiple sessions. The other possible cause may involve the PRP preparation method. In our study, 20 mL of the patient's blood was extracted, double‐spin centrifugation was performed manually without the use of commercial kits, and no activator was added. In Butt et al. study, 9 mL of blood was collected, single‐spin centrifugation was performed with commercial kits, and no activator was added. Consequently, similar to the findings of the studies above, it may be postulated that the manual method and double‐spin centrifugation is the preferred technique for PRP preparation.^19^, ^21^

In a study conducted by Kadry et al., 60 patients were divided into two groups of 30; one group received three sessions of SVF injections at a 1‐month interval, while the other group received PRP under the same conditions.  The hair count and diameter were evaluated 3 months after the last session. In the PRP group, terminal hair count increased from 68.87 ± 34.61% to 79.60 ± 38.27% (a significant increase; *p* = 0.037), and hair diameter increased from 100.56 ± 100.45% to 120 ± 90% (a non‐significant increase; *p* = 0.145) during the follow‐up period. In the SVF group, the mean percentage of terminal hair count increased from 58.30 ± 20.98% to 77.60 ± 7.33% (a highly significant increase; *p* < 0.001), and hair diameter increased from 70 ± 30% to 120 ± 30% (a highly significant increase; *p* < 0.001). Therefore, the hair count increased significantly in both the SVF and PRP groups compared to the baseline, with the SVF group experiencing a greater increase than the PRP group. Significant growth in hair diameter relative to baseline was observed in the SVF group but not in the PRP group.^27^

Similar results were observed in the PRP group of the present study, and a significant increase in the mean hair count was observed. In contrast to the findings of Kadry et al., the increase in hair diameter in the PRP group was also significant. As mentioned previously, this difference may be attributable to the PRP preparation methods, which were the manual method with a double‐spin centrifuge in the current study and the use of a commercial kit with a single‐spin centrifuge in Kadry et al. The differences between the present study and those of Kadry et al. and Butt et al. are summarized in Table 3.

**TABLE 3:** Summary of studies comparing SVF and PRP.

						Hair count/density		Hair diameter		PRP preparation method		
Study	Group type	Number of patients	Number of treatment sessions	Evaluation time	Time of evaluation	Mean ± SD	*p*‐value	Mean ± SD	*p*‐value	Blood volume	Centrifuge method	SVF preparation method
**This study**	**PRP**	9	Three sessions of PRP injection with 1‐month intervals	Two months after the last treatment session	Baseline	15.33 ± 3.12^1^	<0.001	0.06 ± 0.02	0.001	20cc	Manual double‐spin centrifugation	Enzymatic
					Follow‐up	26.78 ± 5.14^1^		0.09 ± 0.02				
	**SVF‐PRP**	9	Two sessions of PRP followed by one session of SVF injection with 1‐month intervals		Baseline	18.67 ± 2.78^1^	<0.001	0.05 ± 0.01	<0.001			
					Follow‐up	26.44 ± 4.19^1^		0.08 ± 0.01				
**Kadry et al**.	**PRP**	30	Three sessions of PRP injection with 1‐month interval	Three months after the last treatment session	Baseline	68.87 ± 34.61%^2^	0.037	0.1 ± 100.45%	0.145	8cc	Commercial kit single‐spin	Enzymatic
					Follow‐up	79.60 ± 38.27%^2^		0.12 ± 90%				
	**SVF**	30	Three sessions of SVF injection with 1‐month interval		Baseline	58.30 ± 20.98%^2^	<0.001	0.07 ± 30%	<0.001			
					Follow‐up	77.60 ± 7.33%^2^		0.12 ± 30%				
**Butt et al**.	**PRP**	11	Two sessions of PRP injection with 1‐month interval	Six months after the last treatment session	Baseline	52.44 ± 9.66^3^	_	_	_	9cc	commercial kit single‐spin	Enzymatic
					Follow‐up	63.72 ± 7.73^3^						
	**SVF&PRP**	11	Two sessions of combined PRP and SVF injection with 1‐month interval		Baseline	37.66 ± 7.43^3^	_	_	_			
					Follow‐up	57.11 ± 7.73^3^						

^1^ Total hair count.

^2^ Terminal hair count.

^3^ Hair density.

In line with several studies,^14^, ^15^, ^22^ no serious side effects were reported in the current study for SVF or PRP injections, with pain during injection and headache being the most common side effects.

## 5 CONCLUSION

Both PRP injection alone and PRP injection with SVF can be effective in treating AGA, resulting in increases in hair count, hair diameter, and physician and patient satisfaction without serious complications.  Additional studies with larger sample sizes and more frequent SVF injections are required to evaluate better the effects of adding SVF to routine PRP treatment and to determine the minimum injection needed to produce a significant difference compared to PRP injection alone. If similar results are found in future studies, it can be suggested that PRP be used earlier in the therapy process and SVF be used later and in multiple sessions.

## CONFLICT OF INTEREST STATEMENT

All the authors declare that there is no conflict of interest for this project.

## ETHICS STATEMENT

All of the collected information was kept confidential and analyzed without a specific name. The present participants in this project were adhered to all Helsinki ethical principles and this study was also registered on the Iranian Clinical Trial Registry (IRCT), registration number: IRCT20210414050963N1. This research was approved by Ethics committee of Iran University of Medical Sciences with the ethic code number; IR.IUMS.FMD.REC.1400.017. The patients were signed informed consent for participating in the study.

## CONSENT FOR PUBLICATION

The authors obtained consent to publish. The current manuscript contains no individual person's data. Therefore, consent to publish is not applicable.

## Data Availability

The data that support the findings of this study are available from the corresponding author, upon reasonable request.
